# Optimized Sensor Data Preprocessing Using Parameter-Transfer Learning for Wind Turbine Power Curve Modeling

**DOI:** 10.3390/s25175329

**Published:** 2025-08-27

**Authors:** Pedro Martín-Calzada, Pedro Martín Sánchez, Francisco Javier Rodríguez-Sánchez, Carlos Santos-Pérez, Jorge Ballesteros

**Affiliations:** 1 Department of Electronics, University of Alcalá, 28805 Alcalá de Henares, Community of Madrid, Spain; pedro.martinc@estudiante.uam.es (P.M.-C.); franciscoj.rodriguez@uah.es (F.J.R.-S.); 2 Department of Signal Theory and Communications, University of Alcalá, 28805 Alcalá de Henares, Community of Madrid, Spain; carlos.santos@uah.es; 3 Instituto Tecnológico y de Energías Renovables (ITER), 38600 Granadilla de Abona, Tenerife, Spain; jballesteros@iter.es

**Keywords:** sensor data preprocessing, sensor data quality, anomaly detection, power curve modeling, optimization, parameter-transfer learning

## Abstract

Wind turbine power curve modeling is essential for wind power forecasting, turbine performance monitoring, and predictive maintenance. However, SCADA data often contain anomalies (e.g., curtailment, sensor faults), degrading the accuracy of power curve predictions. This paper presents a parameter-transfer learning strategy within a preprocessing and modeling framework that jointly optimizes anomaly detection (iForest, LOF, DBSCAN) and WTPC regressors (MLP, RF, GP) via a multi-metric objective adaptable to specific modeling requirements. In the source domain, hyperparameters are explored with randomized search, and in the target domain, transferred settings are refined with Bayesian optimization. Applied to real SCADA from different locations and turbine models, the approach achieves a 90% reduction in optimization iterations and consistently improves target domain performance according to the objective, with no observed loss when comparable source and target turbines differ in site or rated power. Gains are larger for more similar source–target pairs. Overall, the approach yields a practical model-agnostic pipeline that accelerates preprocessing and modeling while preserving or improving fit, particularly for newly installed turbines with limited data.

## 1. Introduction

Wind turbine power curves (WTPCs), which establish the non-linear relationship between wind speed and wind power, play an important role in wind power generation forecasting [1], wind turbine (WT) condition monitoring and performance evaluation [2], and the selection of WTs that closely match the wind regime of a particular site [3]. Such selection is only feasible using the manufacturer’s certified power curve, defined according to existing standards, since operational data are not available until turbines are active.

Generating reliable WTPCs from sensor-based data is challenging because inconsistent observations are common. Typical causes include wind curtailment; control and sensor issues, such as yaw or pitch misalignment, controller delays, and calibration drift; mechanical and communication failures; blade contamination, which reduces aerodynamic efficiency; and atmospheric factors like terrain-induced shear, veer, and air density variations [4,5,6]. Inconsistent data are commonly grouped as *(i)* missing (e.g., sensor or transmission failures), *(ii)* physically irrational data, and *(iii)* outliers [7,8,9]. These inconsistencies degrade predictive accuracy and make WTPCs particularly sensitive to data quality. Most studies therefore adopt a two-step approach.

The first step consists of data preprocessing to mitigate the effect of inconsistent data on model training. Techniques include range and interval filters [10], robust quantile methods [11], density or clustering-based approaches such as DBSCAN [12], image-based cleaning methods [13,14], and anomaly detectors including iForest, LOF, and GMM [15]. Range and quantile filters are simple and fast but may remove valid points near operating boundaries and miss clustered anomalies. By contrast, density and anomaly detection methods such as DBSCAN, iForest, LOF, and GMM capture local structure but require careful tuning and sufficient sample density. Meanwhile, image-based cleaning uses the scatter pattern of wind speed and power at higher computational cost.

The second step involves power curve modeling methods, which can be categorized in various forms. A common way is to consider approaches along two dimensions: *(i)* parametric vs. non-parametric structure [16] and *(ii)* deterministic vs. probabilistic treatment of uncertainty [17]. Parametric formulations (e.g., piece-wise, polynomial, or dynamical power curves) encode aerodynamic insight and are transparent and data-efficient, yet their fixed functional forms limit adaptability when turbines operate under non-nominal regimes [16,18]. Non-parametric learners such as splines, copulas, and artificial neural networks (ANNs) capture site-specific non-linearities with higher accuracy but at the cost of larger SCADA requirements and reduced interpretability [19,20,21]. Deterministic variants yield a single power estimate for each wind speed, offering computational simplicity but no notion of risk, whereas probabilistic counterparts return calibrated predictive distributions, which are an asset for monitoring and forecasting under variable atmospheric conditions [22]. Several studies report that techniques in the non-parametric probabilistic category, notably Gaussian processes (GPs) [23] and full-probability regression models, deliver the lowest errors when data quality is high, albeit with heavier computation [5,24,25].

Among the main areas of WTPC research highlighted in [16], including wind farm development, condition monitoring, data preprocessing and correction, and modeling techniques, this work focuses on data preprocessing, where models are tuned according to the strategies used for power curve generation. This step is essential for developing accurate models for prediction and anomaly detection because *(i)* it enhances data integrity by filtering inconsistent data, ensuring the reliability of historical SCADA records that might otherwise distort the WTPC; *(ii)* it improves model accuracy by boosting predictive performance, particularly when the cleaning process is guided by an objective function based on WTPC behavior; *(iii)* it identifies and isolates anomalies in the data, making it easier to detect and diagnose faults; and *(iv)* it standardizes data formats, facilitating the comparison and integration of datasets across different WTs or time periods. In this regard, automating the preprocessing phase by using optimization strategies based on objective functions can further enhance model performance. These objective functions can incorporate metrics that evaluate the performance of the cleaning algorithms, such as the predictive accuracy of the model trained on clean data or the reduction in training set size. The latter is particularly beneficial for computationally intensive models like GPs, especially when working with large datasets.

However, the preprocessing phase must contend with all types of inconsistent data under heterogeneous operating and environmental conditions [26]. In line with the No Free Lunch principle, no single strategy is universally optimal [27]. Consequently, choices are guided by data and site characteristics, and their impact on WTPC performance is assessed empirically and validated with cross-validation [28], typically by comparing methods such as iForest, LOF, and DBSCAN and by tuning hyperparameters with randomized or grid search when appropriate [29] to balance predictive accuracy and computational cost.

WTPCs are typically characterized by cut-in and cut-out speeds, rated power, and rated wind speed, yet in practice, these values vary with local conditions, which motivates the need for WT-specific nominal curves, a direction that has not been extensively explored.

Manufacturer power curves, which are derived from aerodynamic calculations and validated under standard test conditions, omit site-specific factors, and consequently, performance at the deployment site can differ significantly, leading to discrepancies. Regarding air density, while the manufacturer’s power curve is initially based on reference air density assumptions, it can be recalibrated to reflect local air density, which varies with temperature, atmospheric pressure, and relative humidity. In [30], the authors explored both precorrecting for air density and incorporating it directly into predictive models, particularly with GP modeling, to evaluate their effects on model accuracy. Findings suggest that directly including air density can reduce uncertainty, as precorrection may introduce additional variability due to errors in adjusting wind speed data based on standard air density assumptions. Moreover, local terrain can create unique wind flow patterns that are not represented in the controlled conditions used for validation. This gap between controlled testing environments and real-world conditions motivates the development of improved methods and models to ensure accurate power predictions and efficient wind turbine performance.

Finally, transfer learning, despite its proven effectiveness in various machine learning applications [31], has not been extensively used in WTPC modeling. This is especially true for parameter-transfer approaches where the transferred knowledge is encoded in the shared model parameters or used to inform prior distributions of the model hyperparameters. Transfer learning improves the learning process for a new task by reusing knowledge acquired from a previously encountered task [32]. This paper highlights the considerable potential of transfer learning to address the challenges of inconsistent data in WTPC generation. By applying knowledge gained from one domain (source domain) to enhance model performance in another (target domain), transfer learning can use pre-optimized hyperparameters and thresholds from a well-studied turbine or site to a new, less explored WT. This approach accelerates the convergence of model training, improves predictive accuracy, and reduces the computational cost. Other works, such as those by [33,34], have presented the use of transfer learning in related wind energy contexts. In [33], transfer learning is applied to wind power forecasting, using data from established wind farms to address data scarcity. In [34], transfer learning is used to improve anomaly detection across turbines by fine-tuning thresholds and model layers, rather than re-optimizing transferred parameters. However, these studies focus on adapting specific model components without optimizing the transferred parameters to meet power curve modeling needs.

### Contributions and Paper Organization

The main contributions of this paper are framed within the research subfield of data preprocessing and model selection, and include the following:

1. The methodology automatically estimates optimal hyperparameters for various filtering and anomaly detection strategies, including Isolation Forest (iForest), Local Outlier Factor (LOF) [35], and DBSCAN. This is performed by minimizing a multi-metric objective function that balances the predictive accuracy of models such as GPs, Random Forests (RFs), and ANNs trained on nominal data; the proportion of instances retained after preprocessing; and the resulting variability in the power range.
2. The approach identifies the most important turbine parameters through data analysis, making it independent of specific wind turbine models. These include fundamental WTPC characteristics such as cut-in speed, rated speed, and rated power.
3. The developed strategy for preprocessing sensor-generated SCADA data enables effective knowledge transfer from a wind turbine in the source domain to another in the target domain, supported by an automatic hyperparameter tuning process. In the source domain, an automated cleaning model consisting of filtering and anomaly detection is trained alongside the WTPC model. This results in a robust preprocessing and power curve prediction pipeline tailored to the source turbine. The hyperparameters found to be effective for both data cleaning and WTPC modeling are then transferred to the target turbine, providing a strong initialization. These parameters can be further refined to account for specific characteristics of the target turbine’s operational data, with tuning also performed automatically.
  The benefits of this approach can be summarized as follows: *(i)* it reduces the time and computational resources required to develop accurate preprocessing models for data from new WTs; *(ii)* the automated tuning framework is applicable across different WTs, making the process scalable and consistent; and *(iii)* it ensures that the data cleaning and power curve prediction models remain robust, thereby improving the accuracy of anomaly detection and overall WT performance monitoring.

This paper is organized as follows: Section 1 introduces the importance of wind turbine power curves and the challenges posed by inconsistent SCADA data. Section 2 details the proposed framework and methodology, covering data preprocessing steps such as filtering, anomaly detection, and model training for WTPC generation. Section 3 presents the results, including an initial data quality assessment, the effectiveness of the preprocessing methods, and the impact of parameter-transfer learning across different turbines. Section 4 discusses the findings, emphasizing improvements in data quality, model performance, and computational efficiency enabled by transfer learning.

## 2. Framework and Methodology

**Figure 1:**
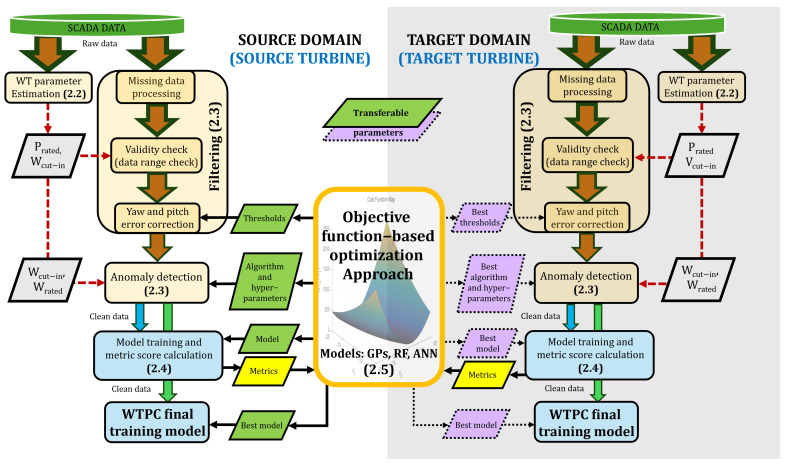
Framework and methodology. Downward arrows represent the flow of data from its acquisition, through preprocessing (filtering and AD), to its use in training the final WTPC model. Red arrows indicate the optional inclusion of parameters. In parentheses, each block includes the subsection detailing the corresponding part of the framework.

This section outlines the framework and methodology employed in this work to preprocess SCADA data and generate reliable wind turbine power curves. Figure 1 illustrates the overall framework and methodology used in this process. The proposed framework integrates advanced filtering and AD techniques based on a parameter-transfer approach to reduce computational effort and to improve WTPC accuracy.

The methodology begins in the source domain with the estimation of wind turbine parameters, which serve as inputs for the filtering and anomaly detection processes. The rated power ($P_{\mathrm{rated}}$) and the cut-in wind speed ($W_{\mathrm{cut}\text{-}\mathrm{in}}$) are used for data range checks, and the cut-in and rated wind speeds ($W_{\mathrm{cut}\text{-}\mathrm{in}}$ and $W_{\mathrm{rated}}$) can guide the choice of anomaly detection algorithms depending on the WTPC region.

The first stage of data preprocessing involves filtering, which includes handling missing values and performing a data range check. Due to inherent delays in control systems and the yaw mechanism itself, turbines cannot remain perfectly aligned with the wind during frequent changes in speed and direction [36]. As a result, yaw errors lead to inconsistent data, which must be removed. Pitch errors, on the other hand, refer to incorrect blade pitch angles that directly affect aerodynamic efficiency and power output. Like yaw errors, pitch errors may arise from control system delays or sensor inaccuracies. However, while yaw errors often generate significant data inconsistencies due to their impact on the alignment of the entire rotor, pitch errors usually cause more localized deviations in output, depending on the severity and duration of the misalignment [37]. Although correcting yaw and pitch error is important, this is not always possible due to the lack of information in some datasets, like the ones considered in this work.

The second stage applies anomaly detection algorithms such as iForest, LOF, and DBSCAN. Its goal is to identify and remove any remaining inconsistencies in the data after filtering, such as anomalous power readings caused by wind curtailment, irrational values from sensor drift, and scattered outliers resulting from communication errors, among others. The third stage involves training a model on the clean data using cross-validation to generate the WTPC and calculate performance metrics. In the fourth stage, the objective function terms are computed to identify the optimal set of hyperparameters. Finally, once the objective function is minimized through an iterative process, the best set of hyperparameters is used to train the final model on the full cleaned dataset, ensuring accurate predictive performance.

In the target domain, the stages mirror those in the source domain: estimation of WT parameters, filtering to address missing values and correct data errors, application of anomaly detection algorithms, and model training. A key advantage, however, is the use of previously optimized parameters from the source domain. This prior knowledge allows the optimization process to converge more rapidly, as the starting point is already near optimal. These values can be directly applied to enable effective anomaly detection on the target wind turbine’s SCADA data. To further improve predictive performance, a refinement step using Bayesian optimization [38] is applied. This approach ensures that the new turbine achieves high accuracy in both anomaly detection and performance prediction, without requiring extensive retraining, thereby making the process efficient and scalable across different turbines and sites.

Parameter-transfer learning presumes comparability between turbines. Therefore, this work assumes a minimal set of compatibility requirements before initializing the target with source parameters. Compatibility is considered along the following three axes. *(i)* Structural: turbines are of the same general type (horizontal axis, variable speed, pitch regulated), with rated power of the same order and similar rotor size, and broadly consistent cut-in, rated, and cut-out ranges. *(ii)* Signal: SCADA data are recorded at the same sampling interval, typically 10 min, and include the same core variables, at least wind speed and active power. *(iii)* Operational: records mainly reflect nominal operation, without persistent curtailment or extended fault periods.

The following subsections describe the SCADA datasets used in this work and provide a detailed explanation of each component of the framework, covering data preprocessing, anomaly detection, and power curve modeling across the source and target domains, with attention to the knowledge transfer between them.

### 2.1. Data Description, Wind Turbine SCADA Datasets, Use Cases of Transfer Learning

Four datasets from four different WTs have been used. They were selected based on their online availability and their compatibility with the transfer learning approach explored in this work. Table 1 summarizes the key characteristics of the datasets, including the location, duration of data collection, SCADA frequency, and various operational parameters such as rated power and the number of instances. The locations of the WTs are Spain (Tenerife), Turkey [39], and the UK [40], with data gathered over a period of 12 months. The SCADA data were recorded at a frequency of 10 min. The rated power of the turbines ranges from 2.05 MW to 3.6 MW. Given the typical setup for SCADA data collection, it is assumed that wind speed was recorded by nacelle-mounted anemometers, as this is standard practice in many turbine datasets that lack detailed metadata [41].

**Figure 2:**
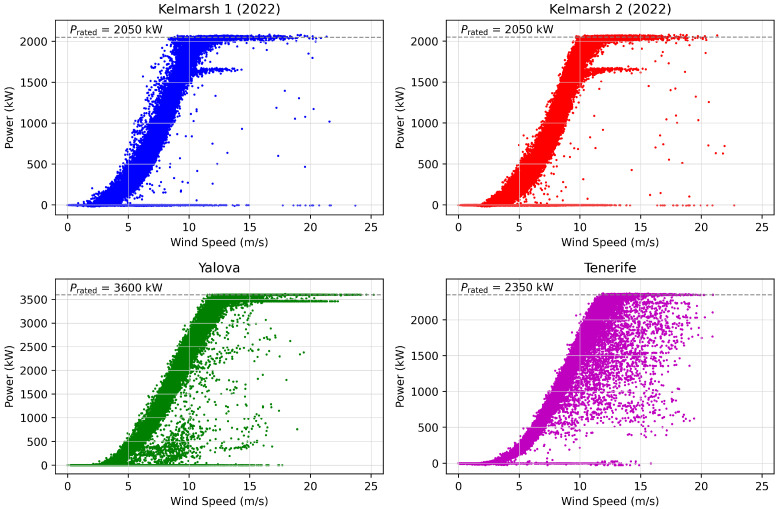
WTPCs under study.

Figure 2 shows the WTPCs for the four wind turbines studied. The figure reveals varying levels of noise across the datasets, with scattered outliers and irregular data points indicating the presence of anomalies and inconsistencies. The power curves for the two turbines located in Kelmarsh are similar, reflecting their identical rated power and shared environmental conditions.

To evaluate the effectiveness of the transfer learning approach, three use cases are considered and shown in Table 2.

The use cases of transfer learning explore different levels of similarity between the source and target domains based on two factors: *(i)* geographical location, distinguishing whether the turbines are in the same wind farm or in different sites, and *(ii)* rated power, considering that all turbines have rated powers of the same order to maintain compatibility. These factors are relevant because location and rated power strongly influence turbine operating conditions, making them suitable for evaluating the applicability of parameter transferability in the proposed approach under varying but realistic scenarios.

In all use cases, the target domain contains only half as much data as the source, as it is limited to a reduced observation period to emulate scenarios with restricted historical data availability, such as when a newly installed or less monitored turbine has limited operational records. In Use Case 1, transfer is applied between two turbines at the same location (Kelmarsh) with the same rated power, using Kelmarsh 1 (2022) as the source and Kelmarsh 2 (H2 2022) as the target. In Use Case 2, the turbines are in different locations but have similar rated power, with Kelmarsh 1 (2022) as the source and Tenerife (November 2023–May 2024) as the target. In Use Case 3, the turbines differ in both location and rated power, with Tenerife (May 2023–May 2024) as the source and Yalova (H2 2018) as the target.

### 2.2. Wind Turbine Characteristics Estimation

When analyzing WT SCADA datasets in depth, it becomes evident that the characteristic parameters provided by manufacturers do not always match the actual behavior of the turbine. As a result, estimating key characteristics such as cut-in and rated wind speeds and rated power directly from SCADA data is necessary, particularly when the values provided by the manufacturer are missing (due to intellectual property restrictions, competitive concerns, or others) or deviate from expected ranges. Moreover, real-world operating conditions can differ from the standard test conditions used by manufacturers, resulting in discrepancies between the theoretical and actual power output. Accurately estimating these parameters under real operating conditions serves several purposes: *(i)* carrying out range checks and filtering out data that fall outside expected ranges, *(ii)* enabling reliable segmentation of the power curve for the potential application of targeted preprocessing techniques and anomaly detection algorithms for each specific region, and  *(iii)* facilitating anomaly detection by setting thresholds based on estimated parameters to identify underperformance or early signs of faults.

The strategy designed in this paper to estimate WT characteristics starts by dividing the power curve into bins following the IEC standard, which uses a bin width of 0.5 m/s for WTPC modeling to avoid distortion from overly wide bins [42]. For each wind speed bin, the corresponding power output is collected from raw SCADA data, which usually contain a considerable amount of inconsistent values. To remove these, the interquartile range (IQR) method is applied, as it is particularly effective in handling noisy datasets due to its robustness to outliers. By focusing on the central 50% of the data, the influence of extreme values is mitigated, which is essential when dealing with noisy or erratic data points, especially those above nominal behavior (≈0 kW) in Region I of the WTPC or below the rated power in Region III.

After applying the inconsistent data removal approach, the median power for each bin is calculated. The median is preferred over the mean because, even after outlier removal, some extreme values may persist due to the inherent variability and potential anomalies in wind turbine data. The median provides a more reliable estimate of the nominal turbine behavior, as it represents the central tendency of the data without being skewed by residual outliers. The resulting median values are then interpolated to approximate the WTPC. Although cubic splines offer greater smoothness, i.e., higher derivative continuity, than Piecewise Cubic Hermite Interpolating Polynomials (PCHIPs), the latter exhibits less overshoot and undershoot [43]. PCHIP is preferred over standard spline interpolation because it preserves the monotonicity of the data. In the context of WTPCs, it is crucial that the interpolation method does not introduce artificial oscillations, which are common with splines. PCHIP ensures that the resulting curve is smooth and follows the natural increase in power output with wind speed, without introducing unrealistic fluctuations.

Finally, an empirically based change rate (e.g., 10.0 kW/(m/s)) is established to determine when the increase in power is significant enough to identify the cut-in speed, and when it stabilizes to mark the rated speed. This threshold, which can be derived from the empirical analysis of several WTs with similar size and rated power, helps quantitatively define the points on the power curve where the turbine starts to generate substantial power (cut-in wind speed) and where it reaches its maximum output (rated wind speed). The choice of a single change rate, rather than two separate thresholds for cut-in and rated speeds, is motivated by the need for consistency and simplicity in the estimation process. While the exact value of the change rate (10.0 kW/(m/s)) may not be universal and could vary with turbine-specific characteristics, using a single threshold ensures a straightforward and uniform approach for identifying these critical speeds.

The rated power is defined as the 99th percentile of the power output. This value represents the level exceeded only 1% of the time, making it a reliable measure of the turbine’s high-performance capabilities. It captures near-maximum operational conditions without being skewed by potential outliers.

The following pseudocode (Algorithm 1) illustrates the method:    
**Algorithm 1:** Estimation of wind turbine characteristic parameters from SCADA data
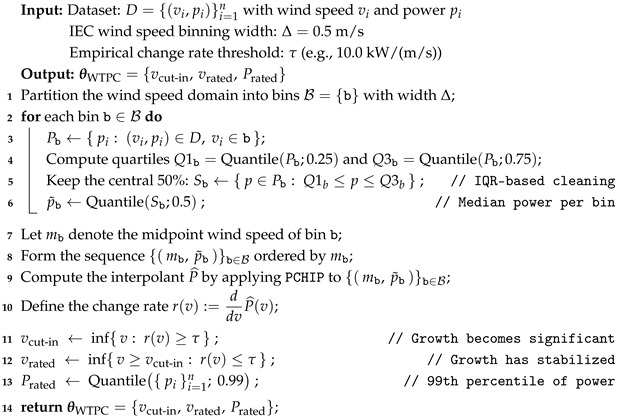


### 2.3. Filtering and Anomaly Detection and Removal: Cleaning Process

The cleaning process consists of two sequential, non-iterative steps, aimed at reducing variance while preserving nominal operation: filtering and anomaly detection and removal. Each step affects a specific type of inconsistent data. Three types are distinguished, encompassing those explained in the introduction of the paper: missing values, erroneous data, and outliers. Missing data cases include rows where the wind speed or power features are empty. Erroneous data include values that are out of range or irrational such as negative power or wind speed, power above 110% of rated power, zero power above cut-in wind speed, or power generation below cut-in wind speed. Finally, outliers are defined as values not classified as either missing or erroneous, including both stacked and scattered types.

As mentioned above, the first stage of data cleaning involves filtering, which includes the elimination of missing values, followed by the removal of erroneous data. If yaw or pitch data are available in both the source and target domain datasets, it can also be used at the end of the filtering stage. This involves defining thresholds that describe turbine behavior incompatible with data consistency. For pitch angles, a threshold of approximately 30º might be considered [15], and yaw error could be quantified at around 2º. The inputs for the filtering block, applicable to both domains, include SCADA data parameters, such as wind speed, power, yaw, and pitch angles (if available), as well as the estimated cut-in wind speed and rated power required for the data range check. The outputs are the filtered wind speed data and their corresponding power values, with inconsistencies removed.

The second stage entails removing outliers using anomaly detection algorithms. Table 3 shows the hyperparameter distributions that have been selected for all datasets considered in this work. If a random seed is required as a hyperparameter, it is always set to 42 as an experimental control decision to ensure reproducibility.

The chosen hyperparameter distributions for each algorithm are designed to cover a broad range of scenarios, ensuring flexibility and robustness in detecting anomalies in WTPC data. They account for typical data characteristics such as variability, density differences, and the proportion of anomalies, making them well suited for this task. For example, robustness to variability and density differences is reflected in the broad ranges of n_estimators in iForest, n_neighbors in LOF, and eps in DBSCAN. Additionally, the contamination ranges for both iForest and LOF represent realistic proportions of anomalies in the dataset.

The inputs of the anomaly detection stage are the filtered wind speed and power from the first stage, and the estimated cut-in and rated wind speeds as optional parameters. Although this work does not implement different anomaly detection algorithms based on the regions of the power curve, these parameters could enable such an approach in future applications. Additionally, the specific anomaly detection algorithm and its hyperparameters, obtained from the optimization stage detailed in Section 2.5, are provided as configuration settings for this stage. The outputs consist of wind speed and corresponding power values labeled as nominal, with anomalies removed, providing a clean dataset for accurate model training in the subsequent stage.

### 2.4. Model Training and Metric Score Calculation

Once the SCADA data have been cleaned, the third stage involves training a model using 5-fold cross-validation to generate the WTPC and calculate performance metrics. The choice of 5-fold cross-validation in this work follows the approach used in other works, such as [44], where it is employed alongside Bayesian optimization (BO) for hyperparameter tuning. This approach balances computational efficiency with predictive accuracy, particularly in models that focus on capturing stable nominal patterns rather than on high-variability forecasting. Applications with greater data fluctuations, such as wind speed or power forecasting, usually use 10-fold cross-validation for its finer-grained error estimation [45]. Thus, for anomaly detection tasks, where identifying deviations from nominal behavior is prioritized, 5-fold cross-validation attains a proper balance. The inputs of this stage are the anomaly-free wind speed and power data, along with the model defined by the optimization algorithm, which selects one of the three regressors mentioned below for power curve fitting. The outputs consist of the calculated performance metrics required by the optimization algorithm. Given the richness of the datasets, non-parametric methods are preferable for modeling, as outlined in Section 1. The models selected cover complementary families and include both deterministic and probabilistic options: RFs from XGBoost (XGBRFRegressor class from xgboost (v2.1.0)) [46], ANNs based on multi-layer perceptrons (MLPRegressor class from scikit-learn (v1.5.2)), and sparse GPs [23] (SparseGPRegression class from GPy (v1.13.2)).

RFs are robust to both outliers and overfitting due to their ensemble nature. The XGBoost-based RF model is optimized for speed and performance, efficiently handling large datasets. Neural networks, particularly MLPs, excel at capturing non-linear relationships in data, approximating a wide variety of functions, and adapting to different types of data distributions. Gaussian processes offer a probabilistic approach to modeling, providing both predictions and uncertainty estimates, which are valuable for performance assessment. Sparse GPs address the scalability issues of standard GPs, making them feasible for large datasets typical in WT analysis. They can model complex, non-linear relationships while incorporating prior knowledge through the choice of kernel functions, enhancing the model’s adaptability to the data.

Evaluating different combinations of hyperparameters for these models is required. However, the number of hyperparameters has been limited due to the high computational cost from trying out different combinations from the AD algorithms and the cross-validation needed to obtain a reasonable performance metric. The random seed was always set to 42 as an experimental control decision to ensure reproducibility. The selected combinations are shown in Table 4.

The selected hyperparameters are aimed at balancing complexity, performance, and regularization. For the XGBRFRegressor, n_estimators (50–200) and min_child_weight (1–10) ensure efficient pattern capture while controlling overfitting by regulating node complexity. As far as the MLPRegressor is concerned, the different variations of hidden_layer_sizes from simple (128) to more complex (32, 64, 32) configurations allow flexibility in capturing data patterns, with the alpha parameter helping to adjust regularization to prevent overfitting. In the case of the sparse GPs, the chosen kernel is an RBF due to its good performance on WTPC data and reduced training time compared with other common kernels [47]. Regarding the number of inducing points, fifty have been selected at random as a balance between accuracy and computational efficiency. Although the parameter space is limited, it attempts to cover both fine detail and broad trend capturing capabilities through the lengthscale values and ensures that the model can handle different levels of data variability and deviations with the variance options.

To evaluate the performance of the WTPC models, a normalized version of the RMSE metric is used. This is calculated in the following way:

(1) $$e_{M}=\frac{\mathrm{RMSE}(X,f)}{P_{\mathrm{rated}}}\times 100\%,$$

with

(2) $$\mathrm{RMSE}\left(X,f\right)=\sqrt{\frac{\sum_{i=1}^{n}{\left(f\left(x_{i}\right)-y_{i}\right)}^{2}}{n}},$$

where *n* is the number of instances in the prediction set, $f(x_{i})$ is the predicted active power value for a specific instance *i* with wind speed $x_{i}$, and $y_{i}$ is the real active power for that instance.

Performance metrics are not the only ones that provide information on the quality of the cleaning strategy. For example, if the goal is to reduce the number of clean points that generate the WTPC, a retention rate metric, i.e., the percentage of data kept, should be considered and minimized. This is calculated as follows:

(3) $$\rho =\frac{N_{a}}{N_{b}}\times 100\%,$$

where $N_{b}$ is the original number of instances in the dataset, and $N_{a}$ is the number of instances after filtering and applying the corresponding AD algorithm.

On the contrary, if it is preferable to maintain as much data as possible, then an elimination rate metric, i.e., the percentage of data removed, should be minimized. There are other metrics that might be considered. Authors in [15] evaluate the change to wind speed interquartile range as an indicator of statistical variability, which can also be measured using the wind power interquartile range. The change in power range refers to the variation in the absolute range of power, bounded between 0 and the rated power. Similarly, the change in wind speed range denotes the variation in the absolute range of wind speed, bounded between 0 and the cut-out speed. Minimizing these changes provides a measure of how many significant power or wind speed values have been preserved while mitigating the impact of anomalous measurements in the original dataset, which could otherwise significantly skew the metric.

The change in power range is worked out in the following way:

(4) $$\Delta R_{p}=\frac{R_{b}-R_{a}}{R_{b}}\times 100\%,$$

where $R_{b}$ is calculated using the formula

(5) $$R_{b}=\min\left(\max\left(Y\right),P_{\mathrm{rated}}\right)-\max\left(\min\left(Y\right),0\right),$$

with *Y* being the power values of all the instances in the original dataset, and $R_{a}$ is determined by using the same equation changing *Y* for the set of power values of the instances that remain after filtering and applying the AD algorithm, $Y_{\mathrm{clean}}$, as follows:

(6) $$R_{a}=\min\left(\max\left(Y_{\mathrm{clean}}\right),P_{\mathrm{rated}}\right)-\max\left(\min\left(Y_{\mathrm{clean}}\right),0\right).$$

### 2.5. Parameters and Hyperparameter Optimization

The core task in both the cleaning stage and WTPC model selection is to solve an optimization problem that estimates the optimal thresholds and hyperparameters. These include thresholds for filtering and the hyperparameters of the anomaly detection strategies and WTPC models, whose values are chosen to minimize an objective function. For simplicity, this set of variables is called parameter space in this paper. As mentioned above, since the size of the parameter space can affect the feasibility of the optimization problem and is subject to the curse of dimensionality, a reduced subset is defined and used in practice.

When tuning the parameter space of machine learning models such as ANN, RF, or GPs, the objective function is typically the validation error. Since this error depends on the entire training process and the resulting model performance, the relationship between the hyperparameters and the validation error is highly complex, allowing the objective function to be treated as a black box. Given the characteristics of black box functions, gradient-based methods cannot be used for optimization. Derivative free optimization (DFO) methods are used instead [48]. Three of these DFO methods are *(i)* grid search, where each point in the parameter space is evaluated and the one that minimizes the objective function is selected; *(ii)* random search, where the points to evaluate are randomly sampled in the parameter space; and *(iii)* Bayesian optimization that uses surrogate models (e.g., Gaussian processes) to model the objective function and selects the next evaluation point from the parameter space based on a probabilistic approach. Other strategies are genetic algorithms and particle swarm optimization. Grid search does not scale to high dimensions because of the curse of dimensionality. Moreover, evaluating the black box objective is expensive even in low dimensions since it first requires a training stage before working out the validation loss. Random search, on the other hand, has proved to be more efficient for hyperparameter optimization [29] in certain machine learning models where the objective function is evaluated on the validation set. The authors proved that random trials are more efficient because not all hyperparameters are equally important; that is, some have little impact on the objective function.

Regarding the objective function, it is advisable to consider multiple metrics, such as those defined in Section 2.4, rather than relying solely on the validation error to select the most suitable AD algorithms and their corresponding hyperparameters for the final goal. In this context, the fourth stage of the proposed framework focuses on computing an objective function to be minimized, defined as a linear combination of some of the previously detailed metrics or others. This work adopts a formulation that rewards cleaner datasets with fewer points that still generate accurate WTPC predictions. It is based on three metrics (normalized RMSE, retention rate, and change in power range), as follows:

(7) $$f_{\mathrm{obj}}=\alpha_{1}\cdot e_{M}+\alpha_{2}\cdot \rho +\alpha_{3}\cdot \Delta R_{p},$$

where $\alpha_{1}=0.6$, $\alpha_{2}=0.1$, and $\alpha_{3}=0.3$ for this work. The coefficients can be adjusted depending on the importance assigned to each metric and their intended purpose.

In this case, normalized RMSE is considered the primary metric, representing 60% ($\alpha_{1}=0.6$) of the objective function. A lower RMSE not only indicates accurate predictions but also evidences the absence of outliers. Retention rate is included to reward datasets with fewer points, suggesting more intensive cleaning. Since a lower RMSE already implies fewer data points, the retention rate accounts for 10% ($\alpha_{2}=0.1$) of the objective function. Lastly, the change in power range is crucial because a lack of points between 0 and the rated power will not generate a complete WTPC. Models trained on datasets with very few points may show a low RMSE, but this alone does not ensure a realistic WTPC. This metric comprises the remaining 30% ($\alpha_{3}=0.3$) of the objective function. This coefficient distribution balances variance reduction (lower $e_{M}$) with coverage (via $\Delta R_{p}$).

The following pseudocode (Algorithm 2) illustrates the method for optimizing the parameter space (Table 3 and Table 4) during the preprocessing and WTPC model selection stages in the source domain. This involves solving an optimization problem to minimize the multi-metric objective function defined above. Due to the complexity of the parameter space and the black box nature of the objective function, a random search approach is employed to efficiently explore it. The algorithm iteratively samples a parameter configuration; i.e., a set of parameter values; evaluates them based on the defined objective function; and selects the optimal set of parameters and trained models that minimize it. For each dataset, AD algorithm, and WTPC regressor model, 30 iterations ($n_{\mathrm{iter}}$) are considered in this work, resulting in a total of 270 runs of the main part of the preprocessing randomized search per dataset. These runs are mutually independent (read-only data access and no cross-run communication) and are therefore embarrassingly parallel. In practice, wall-clock time can be reduced nearly linearly with the number of workers by distributing runs across CPU cores or job arrays. Parallel execution changes only runtime, not results, because randomness is controlled. Accordingly, parallelization can be treated as an implementation detail dependent on machine availability and time constraints, rather than a methodological factor.
**Algorithm 2:** Optimization of the parameter space in the source domain
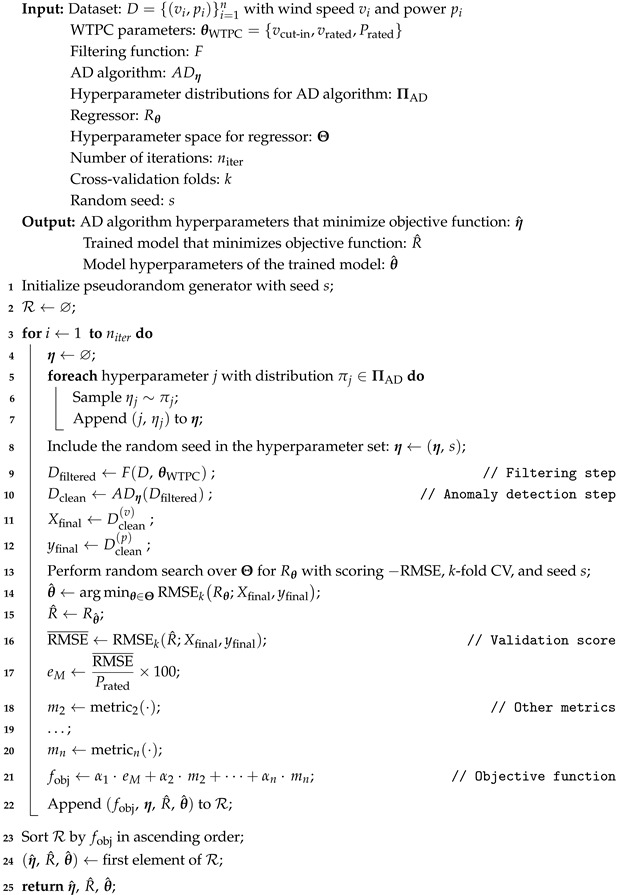


The optimization process in the target domain relies on Bayesian optimization instead of randomized search for several reasons. First, Bayesian optimization can effectively use the knowledge transferred from the source domain, including the best anomaly detection model, its optimized hyperparameters, and the best model for WTPC generation. This prior knowledge allows Bayesian optimization to focus on fine-tuning, reducing the need for a time-consuming random search [49]. Second, Bayesian optimization is computationally more efficient because it models the objective function using surrogate models, such as GPs, which direct the search towards the most localized areas of the parameter space, reducing the number of convergence iterations.

The implementation of Bayesian optimization uses the gp_minimize function from the scikit-optimize (v0.10.2) library. The following pseudocode (Algorithm 3) outlines the evaluation of the objective function in the Bayesian optimization process. As in the source domain, this process involves applying filtering and anomaly detection algorithms to clean the raw data, followed by training a regression model using cross-validation. The performance of the model is then evaluated based on the multi-metric objective function, which combines normalized RMSE and the additional custom metrics mentioned above. The goal is to calculate the value of this objective function, which will guide the Bayesian optimization process for the models in the target domain.
**Algorithm 3:** Optimization step of the parameter space in the target domain**Input**: Dataset: $D={\left\{\left(v_{i},p_{i}\right)\right\}}_{i=1}^{n}$ with wind speed $v_{i}$ and power $p_{i}$         WTPC parameters: $\theta_{\mathrm{WTPC}}=\left\{v_{\mathrm{cut}-\mathrm{in}},v_{\mathrm{rated}},P_{\mathrm{rated}}\right\}$         Filtering function: *F*         AD algorithm: $AD_{\eta }$         Hyperparameters for the AD algorithm: η         Regressor: $R_{\theta }$         Hyperparameters for the regressor: θ         Cross-validation folds: *k*         Random seed: *s***Output**: Float value of the objective function: $f_{\mathrm{obj}}$1Include the random seed in the hyperparameter set: η←(η,s);2$D_{\mathrm{filtered}}\leftarrow F\left(D,\;\theta_{\mathrm{WTPC}}\right)$;                // Filtering step3$D_{\mathrm{clean}}\leftarrow AD_{\eta }\left(D_{\mathrm{filtered}}\right)$;            // Anomaly detection step4$X_{\mathrm{final}}\leftarrow D_{\mathrm{clean}}^{\left(v\right)}$;5$y_{\mathrm{final}}\leftarrow D_{\mathrm{clean}}^{\left(p\right)}$;6$r:={\left\{r_{j}\right\}}_{j=1}^{k}\leftarrow {\mathrm{CV}}_{k}\left(R_{\theta },\;X_{\mathrm{final}},\;y_{\mathrm{final}};s\right)$;7$\bar{\mathrm{RMSE}}\leftarrow \bar{r}=\frac{1}{k}\sum_{j=1}^{k}r_{j}$;               // Validation score8$e_{M}\leftarrow \frac{\bar{\mathrm{RMSE}}}{P_{\mathrm{rated}}}\times 100$;9$m_{2}\leftarrow {\mathrm{metric}}_{2}\left(\cdot \right)$;                     // Other metrics10…;11$m_{n}\leftarrow {\mathrm{metric}}_{n}\left(\cdot \right)$;12$f_{obj}\leftarrow \alpha_{1}\cdot \;e_{M}+\alpha_{2}\cdot \;m_{2}+\ldots +\alpha_{n}\cdot \;m_{n}$;         // Objective function13**return**$f_{obj}$;

## 3. Results and Discussion

This section presents the results of applying the framework to SCADA datasets from four wind turbines located in Tenerife (Spain), Yalova (Turkey), and Kelmarsh (UK). The results provide insights into *(i)* the initial data quality and preprocessing outcomes, *(ii)* the estimation of wind turbine characteristic parameters, *(iii)* the performance of the filtering process and that of different AD algorithms, *(iv)* the optimization of the parameter space and the training and evaluation of WTPC models, and *(v)* the effectiveness of the transfer learning approach in enhancing model performance in the target domain in terms of predictive accuracy and computational efficiency based on the use cases presented in Section 2.1. It is hypothesized that transfer learning will yield greater performance gains when the source and target domains share higher similarity in rated power and location, with the effect expected to diminish as these similarities decrease. Predictive accuracy is assessed through the objective function scores, while computational efficiency is evaluated from the reduction in optimization iterations, the convergence behavior of Bayesian optimization, and the total execution time compared with the source domain.

### 3.1. Initial Data Quality Assessment

Table 5 shows the initial data quality assessment that reveals the extent and type of inconsistent data for each dataset, such as missing values, outliers, and erroneous data points, which are mutually exclusive by the definition used in this work. For this data quality assessment, missing data cases include rows where all features are empty and those missing both power and wind speed values. Erroneous data encompass values that are out of range and irrational values, such as instances where zero wind speed generates power. Outliers are defined as those values identified as such by the interquartile range (IQR) method within standard bins. For this initial analysis, applied equally to all datasets to ensure consistency, none of the anomaly detection algorithms proposed in the framework are used. Using standard methods like IQR provides a straightforward and unbiased way to identify outliers based solely on statistical properties, allowing for a clear baseline assessment of data quality before applying more sophisticated techniques in subsequent stages.

As shown in Table 5, Yalova can be considered the cleanest dataset, with no missing values and a relatively low percentage of erroneous data (5.51%) and outliers (5.01%). In contrast, Tenerife is the most problematic dataset (the noisiest dataset), with the highest percentages of erroneous data (16.67%) and outliers (6.72%), despite having a low percentage of missing values (0.64%). The Kelmarsh 1 and Kelmarsh 2 datasets also exhibit significant data quality issues, particularly in terms of erroneous data and outliers, though Kelmarsh 2 has fewer missing values. Considering that outliers are the most challenging to detect and typically require advanced anomaly detection algorithms, the lower percentage of overall inconsistencies in Yalova makes it the cleanest dataset, while the higher levels of all types in Tenerife, particularly outliers, make it the noisiest dataset.

### 3.2. Estimation of the WT Parameters

Regarding the estimation of wind turbine characteristic parameters, Table 6 combines the real (manufacturer) values, when available, and the estimated values for the cut-in and rated wind speed and the rated power for each dataset. Wind speed estimations are considered comparable to manufacturer values when the absolute difference is less than 0.5 m/s, since the proposed method discretizes wind speed into bins in accordance with the IEC standard [50]. Rated power estimations are regarded as comparable when the relative difference is less than 1% of the manufacturer’s value [51], given that the proposed method defines $P_{\mathrm{rated}}$ as the 99th percentile of the power. Larger deviations are interpreted as case-specific and attributable to the site conditions discussed in Section 2.2.

The combined wind turbine characteristics reveal some interesting insights. The estimated cut-in and rated speeds, as well as the rated power, are generally close to manufacturer specifications. For instance, the cut-in speed for the Kelmarsh 1 dataset is estimated at 2.70 m/s, slightly below the manufacturer’s 3.0 m/s, while the rated speed is very close at 12.21 m/s compared with the manufacturer’s 12.5 m/s. Similarly, the rated power estimations for both the Kelmarsh 1 and Kelmarsh 2 datasets are comparable to those values provided by the manufacturers, with only minor deviations. The Yalova dataset shows a slightly higher estimated cut-in speed at 3.26 m/s compared with 3.0 m/s provided by the manufacturer and a higher rated speed at 14.18 m/s against the expected 13.0 m/s. This suggests potential variations in local wind conditions or turbine performance. The Tenerife dataset also yields estimated values that closely match the manufacturer’s data.

Overall, the results indicate that the estimation method is reliable, providing values that closely match those of the manufacturer, though not identical. These slight variations are expected due to the influence of environmental factors specific to each site, which affect the actual performance of the wind turbines. In situations where the specific wind turbine model is not disclosed due to confidentiality policies, the estimation approach becomes especially valuable to effectively model actual nominal behavior. In this regard, the proximity to the manufacturer values, even in the absence of direct model data, supports the reliability of the method for determining wind turbine parameters under site-specific conditions.

### 3.3. Source Domain Data Cleaning and WTPC Generation, Transferable Prior Knowledge

The following Tables (Table 7, Table 8, Table 9, Table 10) represent the results of the optimization process in the source domain. Each table, corresponding to one of the four datasets, contains nine rows representing the combinations of AD algorithms and WTPC regression models. As mentioned in Section 2.4, a total of 30 iterations are executed for each combination, and the tables show the iteration that yields the best results, i.e., the minimum value of the objective function. In the tables, the combination of AD algorithm and WTPC regression model with the best performance is highlighted in bold. For example, for the Kelmarsh 1 (2022) dataset, the best WTPC regression model and the AD algorithm are MLP and LOF, respectively.

Isolation Forest results in being the most effective method for anomaly detection in the Tenerife dataset, while LOF performs better on the rest, regardless of the regressor used to model the WTPC. This suggests that iForest may be preferable when deviations are more pronounced and dispersed (as in Tenerife), because its random partitioning isolates scattered anomalies effectively, whereas LOF may be preferable when local structure is more regular and well sampled (as in Yalova), since its density-based comparison relies on stable neighborhoods.

Although the DBSCAN results are slightly worse, this AD algorithm does not fall far behind its counterparts. The most likely explanation for its weaker performance is that none of the randomly selected hyperparameter combinations yield results as good as those produced by iForest or LOF, with their respective random combinations. It is also possible that DBSCAN’s use of a single global density criterion leads to difficulties with the WTPC’s non-uniform densities across regimes, which can cause fragmentation of dense nominal regions or the misclassification of sparse nominal zones as noise, resulting in slightly poorer performance.

Across all datasets, MLP proves to be the most effective regressor. However, the differences between the models are marginal. For instance, in the Tenerife dataset, the performance of iForest, rounded to the hundredths, is 8.36 for RF, 8.34 for MLP, and 8.35 for GP. Similar results can be observed in the other tables. Given these small differences, if computational resources are limited, it may be more efficient to focus on a single model rather than all three. The choice of model depends on the advantages that each one brings. For example, RF provides faster results with slightly lower accuracy, while GPs, though slower, offer probabilistic information that could be useful in objective function calculations.

**Figure 3:**
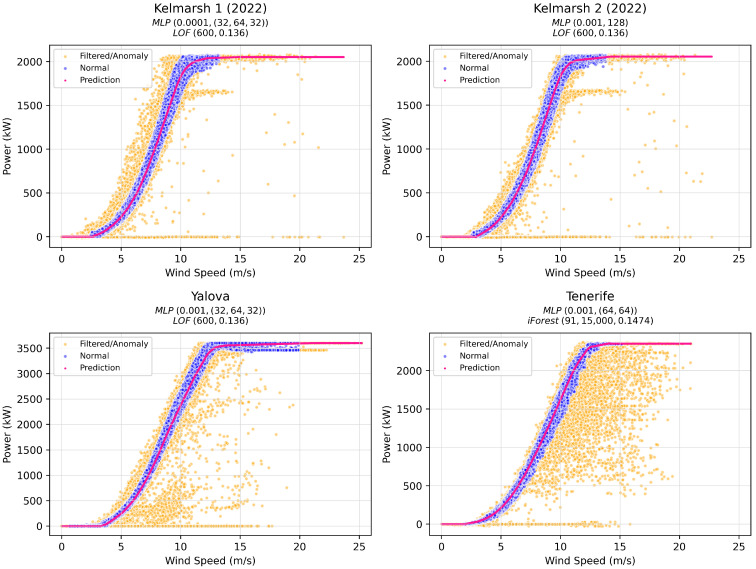
Optimal WTPCs for the source domain.

Figure 3 depicts the WTPC for each of the WTs, after using the models, AD algorithms, and hyperparameters that resulted in the lowest objective function scores, as shown in Table 7, Table 8, Table 9, Table 10. The orange points indicate data that have been removed either through filtering or by applying the AD algorithm. The blue points represent the remaining data, which are considered to reflect nominal turbine behavior. The pink curve depicts the predicted WTPC generated by the selected model. Particularly, this model only applies to the region where there are blue (nominal) data points that are the ones on which the model has been trained. For orange regions outside of this range, i.e., below the cut-in wind speed and above the rated wind speed, the model approximates the power to zero and the rated power, respectively. This approximation arises because areas below cut-in and above rated wind speeds typically have fewer data points and are thus more likely to be flagged as anomalies by the AD algorithms, even though they may not truly be anomalous. However, this is not a problem since the purpose of the WTPC model is to capture the nominal behavior of the turbine. The focus is on modeling the nominal operational performance, with the approximation ensuring that the power curve behaves as expected in areas lacking sufficient data.

It should be noted that visual inspection of the Yalova WTPC in Figure 3 indicates that the rated speed is closer to 14.18 m/s than to 13.0 m/s, further supporting the value presented in Section 3.2 as being more consistent with the observed SCADA data than with the theoretical manufacturer value, possibly due to variations in local wind conditions or turbine performance.

### 3.4. Transfer Learning Evaluation Based on the Use Cases

In this section, the three use cases previously introduced are used to demonstrate the efficacy of the parameter-transfer learning approach in data preprocessing. These use cases illustrate how prior knowledge, particularly the pre-optimized hyperparameters from the source domain, is transferred to the target domain. This approach significantly reduces the need for extensive retraining while accelerating the optimization process even when dealing with turbines in different locations or with varying rated power.

**Figure 4:**
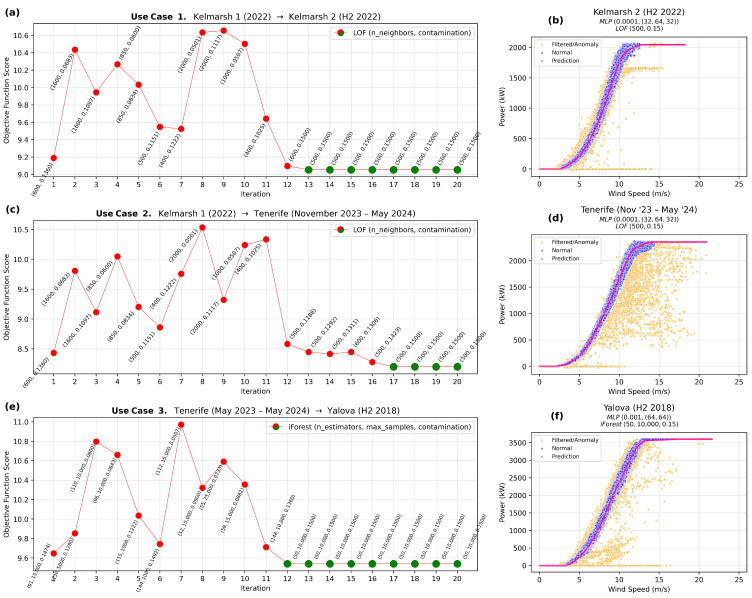
Use cases. Optimal WTPCs for the target domain. (**a**) Use Case 1: BO iterations with convergence by the 13th (green). (**b**) Use Case 1: Optimal WTPC of Kelmarsh 2. (**c**) Use Case 2: BO iterations with convergence by the 17th (green). (**d**) Use Case 2: Optimal WTPC of Tenerife. (**e**) Use Case 3: BO iterations with convergence by the 12th (green). (**f**) Use Case 3: Optimal WTPC of Yalova.

(1) **Use Case 1.** *Source: Kelmarsh 1 (2022)—Target: Kelmarsh 2 (H2 2022).*
  Use Case 1 facilitates transfer learning between two turbines of the same model and therefore rated power, located on the same wind farm. A key aspect is that the target domain, Kelmarsh 2 (H2 2022), uses only half of the data compared with the source domain, Kelmarsh 1 (2022), from which the hyperparameters are transferred.
  As shown in Table 7, the lowest objective function value for Kelmarsh 1 (2022) is achieved with the LOF algorithm (n_neighbors=600, contamination=0.136) and an MLP regressor (alpha=0.0001, hidden_layer_sizes=(32,64,32)). Thus, gp_minimize uses this as the starting point for optimization on Kelmarsh 2 (H2 2022).
  Figure 4a shows that the initial iteration, based on the optimal LOF hyperparameters from the source domain, performs well on the target domain. Rapid convergence is achieved by iteration 13, stabilizing at n_neighbors=500, contamination=0.15, and an objective function value of 9.0539, consistent with a good fit. In comparison, random search on Kelmarsh 2 (second half of 2022) produces a slightly higher value of 9.1894, confirming the effectiveness of the transfer learning approach.
(2) **Use Case 2.** *Source: Kelmarsh 1 (2022)—Target: Tenerife (November 2023–May 2024).*
  Use Case 2 illustrates transfer learning between turbines at different locations but with comparable rated power (2.05 MW vs. 2.35 MW). Similar to Use Case 1, gp_minimize is initialized with LOF hyperparameters (n_neighbors=600, contamination=0.136) and an MLP regressor (alpha=0.0001, hidden_layer_sizes=(32,64,32)).
  Figure 4c shows a clear convergence to the combination of n_neighbors=500 and contamination=0.15 by iteration 17. This results in an objective function value of 8.1994, indicative of a good fit. In comparison, the random search approach on this subset of the Tenerife dataset produces a slightly higher objective of 8.3860.
(3) **Use Case 3.** *Source: Tenerife (May 2023–May 2024)—Target: Yalova (H2 2018).*
  Use Case 3 examines transfer learning between turbines in distinct locations with different rated power (2.35 MW vs. 3.6 MW).
  Table 10 indicates that the lowest objective function value for Tenerife (whole year) is obtained using the iForest algorithm (n_estimators=91, max_samples=15,000, contamination=0.1474) and an MLP regressor (alpha=0.001, hidden_layer_sizes=(64,64)). Accordingly, gp_minimize starts with these parameters when optimizing for Yalova (second half of 2018).
  Figure 4e shows rapid convergence by iteration 12, settling on n_estimators=50, max_samples=10,000, and contamination=0.15, with an objective function value of 9.5394. In comparison, random search on Yalova yields a slightly higher 9.5683.

Figure 4b,d,f represents the final WTPC predictions for Kelmarsh 2, Tenerife, and Yalova, respectively. Despite the reduced data size, the models achieve low objective scores, driven primarily by RMSE, indicating a good fit. Consistently, the predicted power curves (pink) closely align with the distribution of nominal data points (blue). The anomaly detection process effectively filters out mostly inconsistent data (orange), ensuring that the predictions reflect the performance of nominal WT behavior in the target domain.

The results show that transfer learning enhances model performance in target domains, regardless of whether the WTs share similar environments and characteristics or differ in location and rated power. Based on the objective function values, predictions in the target domains consistently show improvements over those in the source domains, highlighting the effectiveness of the transferred knowledge. The initial hypothesis was that transfer learning would yield greater performance gains when the similarity between the source and target domains is higher. This expectation is largely confirmed, as the differences in objective function values between source and target domains are greater for the first two use cases (0.1355 and 0.1866) than for the third (0.0289). However, since the difference in the third use case is not statistically significant, unlike in the other two, this suggests that a larger gap in rated power between turbines has a stronger impact than differences in geographical location.

Regarding the time required for the computations in the source domain, Table 11 presents the average time per iteration and dataset for each combination of WTPC regression model and AD algorithm (30 iterations per combination). Additionally, the computations were conducted on an 11th Gen Intel(R) Core(TM) i7-1165G7 @ 2.80GHz (Intel, Santa Clara, CA, USA).

Table 12 shows the time required for optimization in the source domain compared with the target domain across all use cases. In all of them, transfer learning significantly reduces model training time, cutting it from hours to a single minute (from 270 iterations using randomized search on model hyperparameters to just 20 iterations with BO given pre-determined hyperparameter combinations). It is important to note that GPs account for over 90% of the time in the source domain, whereas they are not used in the target domain, where MLPs performed best. Therefore, although the absolute values may vary in a different context, the time reduction remains evident.

## 4. Conclusions and Future Directions

This paper introduces a robust and flexible framework and methodology for SCADA data cleaning and WTPC generation. It effectively addresses the challenges posed by inconsistent sensor-generated SCADA data, including anomalies such as missing values, sensor measurement errors, and outliers resulting from sensor faults or operational malfunctions. By integrating advanced anomaly detection algorithms, such as iForest, LOF, and DBSCAN, with automatic hyperparameter optimization, the proposed approach improves the accuracy of WTPC models while minimizing the computational burden in terms of number of iterations.

A significant contribution of this work is the successful application of transfer learning across different wind turbines and farm locations. By transferring pre-optimized parameters and hyperparameters from a source WT to a target WT, the framework demonstrates its effectiveness in improving model convergence and predictive accuracy. Moreover, the approach reduces the number of iterations required by approximately 90% (from 270 in the source domain to 20 in the target domain) and can potentially be higher depending on the number of AD–regressor combinations and BO iterations.

The evaluation of three use cases illustrates the adaptability of the framework to different operational scenarios, including turbines in the same wind farm as well as turbines operating in other geographical regions. In all cases, transfer learning accelerates the optimization process while maintaining or improving model performance, thereby reducing the need for extensive retraining.

However, certain limitations are recognized. First, while the model effectively captures nominal turbine performance, it does not fully address all contextual anomalies due to the absence of detailed state and substate data, which may impact accuracy under specific operational scenarios, such as grid service participation. Another area for future development is the implementation of incremental anomaly detection, which would enable the model to adapt to gradual shifts in turbine performance over time, such as those caused by wear or changing environmental conditions. This approach would enhance the ability of the model to distinguish between short-term anomalies and long-term changes in nominal behavior, improving its utility for long-term monitoring.

Future work will aim to address these limitations by incorporating a more granular approach to contextual anomaly detection, expanding validation methods, and implementing incremental anomaly detection to refine the adaptability and reliability of the model across diverse operational conditions. In particular, contextual AD could condition detectors on available context (state and substate, curtailment or grid service flags, WTPC region), selecting thresholds locally to reduce false positives. Incremental AD could use rolling windows with drift triggers and warm-started updates to adapt gradually without full retraining [52,53,54]. Finally, future work will also include a systematic sensitivity analysis to quantify the influence of hyperparameters, the choice of AD algorithm, and site-specific conditions on the model performance. While the results confirm improvements in computational efficiency and iteration reduction, a more detailed assessment of sensitivity across more heterogeneous scenarios remains an open direction.

## Figures and Tables

**Table 1:** WTs and datasets used in this paper.

Wind Turbine	WT1	WT2	WT3	WT4
**Location**	Spain (Tenerife)	Turkey (Yalova)	UK (Kelmarsh 1)	UK (Kelmarsh 2)
**Start/end period**	16 May 2023–14 May 2024	1 January 2018–1 January 2019	1 January 2022–31 December 2022	1 January 2022–31 December 2022
**SCADA duration**	12 months	12 months	12 months	12 months
**SCADA frequency**	10 min	10 min	10 min	10 min
**Rated power**	2.35 MW	3.6 MW	2.05 MW	2.05 MW
**Instances**	52,507	50,530	52,560	52,560

**Table 2:** Use cases of transfer learning depending on the similarity between location and $P_{\mathrm{rated}}$.

Use Case	Source Domain	Target Domain
**1** (=loc, =$P_{\mathrm{rated}})$	Kelmarsh 1 (2022)	Kelmarsh 2 (H2 2022)
**2** (≠loc, ≈$P_{\mathrm{rated}})$	Kelmarsh 1 (2022)	Tenerife (Nov. ’23–May ’24)
**3** (≠loc, ≠$P_{\mathrm{rated}})$	Tenerife (May ’23–May ’24)	Yalova (H2 2018)

**Table 3:** Hyperparameter distributions and values for the anomaly detection algorithms.

Algorithm	Hyperparameter	Distribution/Values
iForest	n_estimators	Random integer between 50 and 150 (inclusive).
	max_samples	{1000, 5000, 10,000, 15,000, 20,000, 25,000}.
	contamination	Uniform distribution between 0.05 and 0.15.
LOF	n_neighbors	{400, 500, 600, 700, 850, 1000, 1300, 1600, 2000}.
	contamination	Uniform distribution between 0.05 and 0.15.
DBSCAN	eps	Uniform distribution between 0.01 and 0.10.
	min_samples	Random integer between 20 and 50 (inclusive).

**Table 4:** WTPC regression models and their hyperparameters.

Model	Hyperparameter	Values
Random Forest (XGBRF Regressor)	n_estimators	50, 100, 150, 200
	min_child_weight	1, 5, 10
MLP Regressor (MLPRegressor)	hidden_layer_sizes	(32, 32), (32, 64), (64, 64), (128), (32, 64, 32)
	alpha	0.0001, 0.001
Sparse GP Regressor (SparseGPRegression) (ind.points = 50, kernel = RBF)	lengthscale	1, 5
	variance	1000, 2000

**Table 5:** SCADA data quality (initial assessment).

Dataset	Total Size	Missing Values (Count/%)	Erroneous Data (Count/%)	Outliers (Count/%)
Kelmarsh 1 (2022)	52,560	554 (1.05%)	6693 (12.73%)	1689 (3.21%)
Kelmarsh 2 (2022)	52,560	72 (0.14%)	7134 (13.57%)	930 (1.77%)
Yalova	50,530	0 (0.00%)	2785 (5.51%)	2531 (5.01%)
Tenerife	52,507	335 (0.64%)	8751 (16.67%)	3526 (6.72%)

**Table 6:** Summary of the SCADA data characteristics for the four WTs. The manufacturers’ values are presented alongside the estimated (est.) values obtained using the proposed method for comparison.

Dataset	Cut-In Speed (Manufacturer ‖ est.)	Rated Speed (Manufacturer ‖ est.)	Rated Power (Manufacturer ‖ est.)
Kelmarsh 1 (2022)	3.0 m/s ‖ 2.70 m/s	12.5 m/s ‖ 12.21 m/s	2050 kW ‖ 2052.72 kW
Kelmarsh 2 (2022)	3.0 m/s ‖ 2.78 m/s	12.5 m/s ‖ 12.24 m/s	2050 kW ‖ 2054.91 kW
Yalova	3.0 m/s ‖ 3.26 m/s	13.0 m/s ‖ 14.18 m/s	3600 kW ‖ 3603.63 kW
Tenerife	2.0 m/s ‖ 2.10 m/s	14.0 m/s ‖ 14.14 m/s	2350 kW ‖ 2353.27 kW

**Table 7:** Source domain optimization results for Kelmarsh 1 (2022).

Model	Model Hyperparameters ^1^	AD Algorithm with Hyperparameters ^2^	Objective Function Score
	(1, 200)	iForest (67, 15,000, 0.1442)	9.4900
RF	(1, 150)	LOF (600, 0.136)	9.4409
	(1, 200)	DBSCAN (0.0256, 36)	9.7632
	(0.001, (32, 32))	iForest (51, 15,000, 0.1439)	9.4377
MLP	**(0.0001, (32, 64, 32))**	**LOF (600, 0.136)**	**9.3757**
	(0.001, (32, 64))	DBSCAN (0.0256, 36)	9.6712
	(1, 2000)	iForest (67, 15,000, 0.1442)	9.4777
GP	(5, 1000)	LOF (600, 0.136)	9.4256
	(5, 2000)	DBSCAN (0.0256, 36)	9.7516

^1^ RF (min_child_weight, n_estimators), MLP (alpha, hidden_layer_sizes), GP (lengthscale, variance); ^2^ iForest (n_estimators, max_samples, contamination), LOF (n_neighbors, contamination), DBSCAN (eps, min_samples).

**Table 8:** Source domain optimization results for Kelmarsh 2 (2022).

Model	Model Hyperparameters ^1^	AD Algorithm with Hyperparameters ^2^	Objective Function Score
	(1, 50)	iForest (51, 15,000, 0.1439)	9.6789
RF	(1, 200)	LOF (600, 0.136)	9.5913
	(1, 100)	DBSCAN (0.0256, 36)	10.3360
	(0.0001, (32, 64))	iForest (57, 10,000, 0.1409)	9.6207
MLP	**(0.001, (128))**	**LOF (600, 0.136)**	**9.5766**
	(0.001, (32, 64))	DBSCAN (0.0256, 36)	10.2679
	(1, 2000)	iForest (51, 15,000, 0.1439)	9.6687
GP	(5, 1000)	LOF (600, 0.136)	9.5812
	(5, 2000)	DBSCAN (0.0256, 36)	10.3269

^1^ RF (min_child_weight, n_estimators), MLP (alpha, hidden_layer_sizes), GP (lengthscale, variance); ^2^ iForest (n_estimators, max_samples, contamination), LOF (n_neighbors, contamination), DBSCAN (eps, min_samples).

**Table 9:** Source domain optimization results for Yalova.

Model	Model Hyperparameters ^1^	AD Algorithm with Hyperparameters ^2^	Objective Function Score
	(1, 100)	iForest (91, 15,000, 0.1474)	9.4478
RF	(1, 150)	LOF (600, 0.136)	9.4213
	(1, 200)	DBSCAN (0.0142, 38)	9.7378
	(0.0001, (32, 64, 32))	iForest (91, 15,000, 0.1474)	9.3788
MLP	**(0.001, (128))**	**LOF (600, 0.136)**	**9.4346**
	(0.001, (32, 64))	DBSCAN (0.0142, 38)	9.9046
	(1, 2000)	iForest (91, 15,000, 0.1474)	9.4346
GP	(1, 2000)	LOF (600, 0.136)	9.4096
	(1, 2000)	DBSCAN (0.0142, 38)	9.7408

^1^ RF (min_child_weight, n_estimators), MLP (alpha, hidden_layer_sizes), GP (lengthscale, variance); ^2^ iForest (n_estimators, max_samples, contamination), LOF (n_neighbors, contamination), DBSCAN (eps, min_samples).

**Table 10:** Source domain optimization results for Tenerife.

Model	Model Hyperparameters ^1^	AD Algorithm with Hyperparameters ^2^	Objective Function Score
	(1, 200)	iForest (91, 15,000, 0.1474)	8.3642
RF	(1, 200)	LOF (2000, 0.1466)	8.4856
	(1, 50)	DBSCAN (0.0159, 23)	8.6268
	**(0.001, (64, 64))**	**iForest (91, 15,000, 0.1474)**	**8.3443**
MLP	(0.0001, (32, 64))	LOF (2000, 0.1466)	8.4772
	(0.0001, (32, 64))	DBSCAN (0.0159, 23)	8.6140
	(1, 2000)	iForest (91, 15,000, 0.1474)	8.3537
GP	(5, 1000)	LOF (2000, 0.1466)	8.4718
	(1, 1000)	DBSCAN (0.0159, 23)	8.6124

^1^ RF (min_child_weight, n_estimators), MLP (alpha, hidden_layer_sizes), GP (lengthscale, variance); ^2^ iForest (n_estimators, max_samples, contamination), LOF (n_neighbors, contamination), DBSCAN (eps, min_samples).

**Table 11:** Source domain average times per iteration.

Model	AD Algorithm	Average Time per Iteration
RF	iForest	6.03 s
	LOF	13.87 s
	DBSCAN	5.67 s
MLP	iForest	17.57 s
	LOF	25.27 s
	DBSCAN	16.9 s
GP	iForest	11 min 28 s
	LOF	20 min 53 s
	DBSCAN	10 min 56 s

**Table 12:** Execution times for source and target domains for each use case.

Use Case	Source Domain Time	Target Domain Time
**1**	22 h 22 min 52 s	59.3 s
**2**	21 h 50 min 4 s	52.7 s
**3**	22 h 46 min 14 s	31.2 s

## Data Availability

The authors do not have permission to share data of the Tenerife wind turbine. The datasets of the other turbines can be found in [39,40].

## Abbreviations

The following abbreviations are used in this article:

AD	Anomaly Detection
ANN	Artificial Neural Network
BO	Bayesian Optimization
CPU	Central Processing Unit
DBSCAN	Density-Based Spatial Clustering of Applications with Noise
DFO	Derivative Free Optimization
GMM	Gaussian Mixture Modeling
GP	Gaussian Process
H2	Second Half of a Calendar Year
IEC	International Electrotechnical Commission
iForest	Isolation Forest
IQR	Interquartile Range
LOF	Local Outlier Factor
MLP	Multilayer Perceptron
PCHIP	Piecewise Cubic Hermite Interpolating Polynomial
RBF	Radial Basis Function (kernel)
RF	Random Forest
RMSE	Root Mean Squared Error
SCADA	Supervisory Control and Data Acquisition
WT	Wind Turbine
WTPC	Wind Turbine Power Curve
XGBoost	eXtreme Gradient Boosting

## Symbols

The following symbols are used in this article:

$\alpha_{i}$	metric weights of the objective function
$\Delta R_{p}$	change in power range
$e_{M}$	prediction error as average of $P_{\mathrm{rated}}$
*f*	power prediction function
$f_{\mathrm{obj}}$	objective function
$f(x_{i})$	predicted power for instance *i* with wind speed *x*
*n*	number of instances in test set
$N_{b}$, $N_{a}$	number of instances before and after AD
$P_{\mathrm{rated}}$	rated power of the wind turbine
ρ	retention rate
$R_{b}$, $R_{a}$	power range before and after AD
$W_{\mathrm{cut}\text{-}\mathrm{in}}$	cut-in wind speed of the wind turbine
$W_{\mathrm{rated}}$	rated wind speed of the wind turbine
*X*	test set
$x_{i}$	wind speed value for instance *i*
*Y*	power values of the dataset
$Y_{\mathrm{clean}}$	power values of instances after AD
$y_{i}$	actual power value for instance *i*

## Units

The following units are used in this article:

kW	kilowatts
m/s	meters/second
MW	megawatts
º	degree
